# Retraction Note: Emerging insights into the role of IL-1 inhibitors and colchicine for inflammation control in type 2 diabetes

**DOI:** 10.1186/s13098-025-01596-w

**Published:** 2025-01-20

**Authors:** Jianbin Guan, Haimiti Abudouaini, Kaiyuan Lin, Kaitan Yang

**Affiliations:** 1 Honghui-Hospital, Xi’an Jiaotong University, Xi’an, 710054 Shaanxi China; 2 Truma Rehabilitation Department, Honghui-Hospital, Xi’an Jiaotong University, Xi’an, 710054 Shaanxi China

**Retraction Note: Diabetology & Metabolic Syndrome (2024) 16:140**

10.1186/s13098-024-01369-x

The Editors-in-Chief have retracted this article because it shows significant overlap with an unpublished manuscript under review at another journal. Jianbin Guan, Haimiti Abudouaini and Kaitan Yang agree with this retraction. Kaiyuan Lin has not responded to correspondence from the Publisher about this retraction.

